# Matrine alleviates cisplatin‐induced acute kidney injury by inhibiting mitochondrial dysfunction and inflammation via SIRT3/OPA1 pathway

**DOI:** 10.1111/jcmm.17398

**Published:** 2022-06-01

**Authors:** Lu Yuan, Jingchao Yang, Ying Li, Longhui Yuan, Fei Liu, Yujia Yuan, Xiaochi Tang

**Affiliations:** ^1^ The First People's Hospital of Shuangliu District, Airport Hospital of West China Hospital West China Hospital, Sichuan University Chengdu China; ^2^ Key Laboratory of Transplant Engineering and Immunology, NHFPC West China Hospital, Sichuan University Chengdu China

**Keywords:** acute kidney injury, Matrine, mitochondria dysfunction, OPA1, SIRT3

## Abstract

Cisplatin is extensively used to treat malignancies. However, its clinical use is always limited due to the serious side effects, especially the nephrotoxicity. Matrine (MAT), a tetracyclic quinolizine alkaloid found in sophora genus, exerts multiple pharmacological roles, including anti‐oxidative stress, anti‐inflammation and anti‐apoptosis, but the role of MAT on acute kidney injury (AKI) has not been evaluated. Here, we found that MAT potently inhibited cell injury induced by cisplatin in HK2 cells in vitro, which was associated with the inhibition of oxidative injury and NF‐κB‐mediated inflammation. Moreover, MAT treatment could activate the SIRT3/OPA1 axis and subsequently suppress the mitochondrial fragmentation and improve mitochondrial function. More importantly, SIRT3 knockdown suppressed the deacetylation of OPA1, which blocked the protective role of MAT on cisplatin‐induced cell injury. In vivo, MAT treatment alleviated renal dysfunction, histological damage and inflammation induced by cisplatin in mice. Furthermore, consistent with the founding in vitro, MAT also activated SIRT3‐mediated deacetylation of OPA1 and alleviated mitochondrial dysfunction in AKI mice. Our study proved that MAT protected against cisplatin‐induced AKI by synergic anti‐oxidative stress and anti‐inflammation actions via SIRT3/OPA1‐mediated improvement of mitochondrial function, suggesting that MAT may be a novel and effective strategy for AKI.

## 1 INTRODUCTION

As a common chemotherapeutic agent, cisplatin is extensively used for many years to treat various solid tumours such as lung, testis and ovarian cancers. However, because of the excessive accumulation in renal tubular cells, cisplatin‐induced acute kidney injury (AKI) greatly limits its use.^1^ In one study, 20%–40% of patients following the administration of cisplatin developed into moderate or severe renal injury.^2^

The pathological mechanism of cisplatin‐induced AKI is complicated. As previously reported, after renal uptake, cisplatin is hydrolysed into a positively charged electrophile and accumulates within the negatively charged mitochondrial membrane, which in turn disrupt its membrane potential. As a consequence, the depolarized mitochondria induce mtROS generation and abnormal pathological structure in mitochondria by suppressing mitochondrial energetic metabolism and ATP production.^3^ Moreover, along with the decrease in antioxidant capacity in AKI, the subsequent oxidative stress due to burst of mtROS cause direct oxidative damage to DNA, proteins and lipids, thereby inducing inflammation by activating toll‐like receptors (TLRs) and the NLRP3 inflammasome pathway.^4^, ^5^ In addition, in cisplatin‐induced AKI mice, damaged mitochondria‐derived mitochondrial DNA (mtDNA) was released into the cytosol, which also causes inflammation via activation of the cyclic GMP‐AMP synthase (cGAS) stimulator of interferon genes (STING) pathway.^6^ Thus, mitochondrial dysfunction caused by impaired mitochondrial redox balance and the subsequence of inflammation are the critical reasons for AKI.^7^, ^8^ Conversely, mitochondria‐targeted strategies to improve mitochondrial function have been shown to inhibit tubular cell death and systemic inflammation, and maintain renal function in AKI.^9^, ^10^, ^11^ Therefore, an effective strategy for preserving healthy mitochondria and inhibiting of inflammation is essential for the homeostasis of kidney in AKI.

Matrine (MAT), a tetracyclic quinolizine alkaloid found in sophora genus, exerts multiple pharmacological roles, such as anti‐oxidative stress, anti‐inflammation and anti‐apoptosis,^12^, ^13^ It has been demonstrated that MAT could alleviate several diseases, including acute lung injury, acute liver injury and cardiac injury.^14^, ^15^, ^16^ Recently, MAT has been demonstrated to attenuate experimental autoimmune encephalomyelitis (EAE) in vivo via enhancing mitophagy, inhibiting oxidative injury and inflammation.^12^ whereas the effects of MAT on acute kidney injury induced by cisplatin have not yet been studied.

Considering the role of MAT in regulating mitochondrial function and inflammation response, we evaluated the effects of MAT on cisplatin‐caused acute kidney injury in our study.

## 2 MATERIALS AND METHODS

### 2.1 Cell culture and treatment

The human proximal tubular epithelial cell line HK2 cells were obtained from the American Type Culture Collection (Manassas) and cultured in complete medium, which is composed of DMEM medium (Invitrogen) supplemented with 10% FBS (Gibco), 100 U/ml penicillin and 100 μg/ml streptomycin at 37°C in 5% CO_2_ in a humidified incubator. To evaluate the effects of MAT on cisplatin‐induced cell injury, cells incubated with 10 μM cisplatin (HY‐17394; MCE) for 48 h were treated with or without matrine (400 μΜ). Matrine (≥99% by HPLC, HY‐N0164) was purchased from MCE and prepared as previously described.^17^, ^18^ Briefly, MAT was dissolved in warm PBS and used as stock solution (40 mM), and the stock solution was further diluted to different concentrations with PBS.

### 2.2 Cell viability assay

HK2 cells were seeded in a 96‐well plate. After the treatment, cell viability was detected by cell counting kit‐8 (CCK‐8; CK04; Dojindo Laboratories). Briefly, CCK‐8 solution was added and incubated with the cells at 37°C for 2 h. Then, the absorbance at 450 nm was measured with a microplate reader (BioTek Instruments Inc.). Cell viability was calculated by normalizing the optical density of the experimental group to that of the control group.

### 2.3 Cell apoptosis analysis

Apoptosis was measured via Annexin V‐FITC/PI kit (556,507; BD Biosciences) as previously described.^19^ After the treatment, cells were collected with trypsin without EDTA and washed with cold phosphate‐buffered saline (PBS). Then, cells diluted to 1 × 10^6^ cells/ml with annexin V‐binding buffer (100 L) were added with 5 μl Annexin V‐FITC and PI for 15 min at 4°C in the dark. After the incubation, cell apoptosis was analysed by flow cytometry (Beckman Coulter).

### 2.4 ROS detection

For intracellular ROS and mitochondrial ROS (mtROS) detection, cells were stained with DCFH‐DA (10 μM; 4091‐99‐0; Sigma‐Aldrich) or MitoSOX™ Red (2.5 μM; M36008; Thermo Fisher Scientific) for 15 min at 37°C in the dark. Then, cells were observed by fluorescence microscope and analysed by Image J software.

### 2.5 Evaluation of MDA content and CAT activity

We measured the MDA content and CAT activity in the cells via MDA (S0131; Beyotime Biotechnology) and CAT Assay Kit (S0051; Beyotime Biotechnology), following the manufacturer’ s protocol.

### 2.6 Mitochondrial membrane potential (Δψm) assay

Cells were incubated with 100 nM TMRM (T668; Thermo Fisher Scientific) at 37°C for 30 min. After that, cells were washed with PBS for twice and then observed by fluorescence microscope. For quantification, the mean fluorescence intensity was analysed using Image J software.

### 2.7 Mitochondrial morphology

To observe the mitochondrial morphology, cells were incubated with Mito‐Tracker Green (C1048; Beyotime Biotechnology) at 37°C for 30 min, and then, the mitochondrial morphology was visualized by confocal microscope.

### 2.8 ATP measurement

The ATP was determined by ATP Assay Kit (S0026; Beyotime Biotechnology). For ATP measurement, 100 μl supernatant was mixed with an equal volume of luciferase reagent; then, luminescence was measured by SpectraMax M5 MultiMode Microplate Reader.

### 2.9 Western blot analysis

Western blot was performed as previously described.^20^ Proteins from frozen kidney tissue and cultured HK2 cells were extracted using RIRA buffer (P0013C; Beyotime Biotechnology) with PMSF (8553; Cell Signaling Technology) for 1 h, and the protein concentration was determined using the BCA protein assay kit (C503051; Sangon Biotech). Then, samples containing 30 μg of protein were separated by SDS‐PAGE and transferred PVDF to membranes (IPVH00010; Millipore). The membranes were blocked with 5% non‐fat milk powder dissolved in 1 × PBS containing 0.05% Tween‐20 and then incubated with primary antibodies overnight (12 h) at 4 °C (BAX, 2772S, CST; Bcl‐2, A2212, ABclonal; TFAM, ab131607, Abcam; PGC‐1α, ab54481, Abcam; NDUFS4, A13519, Abclonal; ATP5a, A5884, Abclonal; ACTIN, AC026, Abclonal; p‐NF‐κB, AP0125, Abclonal; NF‐κB, A6667, Abclonal). After washing three times with TBST, the membranes were incubated with secondary antibodies for 1 h at room temperature. Finally, protein bands were detected by enhanced chemiluminescence kit (WBKLS0500) and quantified by Image J software.

### 2.10 Immunoprecipitation

For CO‐IP, 1 mg of protein in 100 μl of lysis buffer was pre‐cleared for 1 h at 4°C with protein A + G agarose (P2012; Beyotime Biotechnology). After that, rabbit anti‐SIRT3 (D22A3; Cell Signaling Technology) or mouse anti‐OPA1 (ab119685; Abcam) was added to the sample and incubated for overnight at 4°C. Then, the complex was added with 20 μl of protein A + G agarose at 4°C for 4 h. After incubation and washing, immunoprecipitates were boiled in SDS/PAGE loading buffer and subjected to Western blotting analysis. To detect the acetylated OPA1, the protein was incubated with 1 μg of anti‐OPA1 overnight, and then, the samples were measured using the rabbit antibody of acetylated lysine (9441; CST) by Western blotting.

### 2.11 SIRT3 siRNA transfection

Scrambled siRNA and SIRT3 siRNA (sense: 5′‐ ACUCCCAUUCUUCUCUAC dTdT‐3′; antisense: 5′‐UGAGGGUAAGAAGAGAUG dTdT‐3′) were chemically synthesized by Ribobio. Transfection experiments of HK2 cells were performed with jetPRIME transfection reagent (101,000,001; Polyplus Transfection) according to the manufacturer's instructions.

### 2.12 Immunofluorescence (IF) staining

4% paraformaldehyde‐fixed cells or kidney frozen sections were permeated with 0.3% Triton for 15 min and incubated with NF‐κB antibody (4764S, CST) or ATP5a antibody at 4°C overnight, then incubated with FITC or TRITC‐conjugated secondary antibodies for 1 h at 37°C. Nuclei were stained with DAPI (C9819; Sigma‐Aldrich). For observation, images were captured by fluorescent microscope, and the nuclear/cytoplasm ratio of NF‐κB was analysed by Image J software.

### 2.13 Animal model

Male C57BL/6 mice (8 weeks old) were purchased from Chengdu Dossy Experimental Animal Co. Ltd. and maintained in SPF room at 25 ± 1°C with a 12‐h light/dark cycle and accessed standard food and water ad libitum. Animal experiments were approved and carried out in accordance with the guidelines of Animal Care and Use Committee of Sichuan University. To evaluate the effects of matrine (MAT) on cisplatin‐induced acute kidney injury (AKI), mice were assigned to 4 groups (normal control, NC: *n* = 8; MAT, *N* = 8; cisplatin, *n* = 8; and cisplatin + MAT, *n* = 8). Specifically, mice were intraperitoneally (i.p.) injected with cisplatin (20 mg/kg, single injection, MCE). For MAT treatment, MAT was diluted with normal saline to concentration (5 mg/ml) and used immediately, and MAT was injected (i.p. 5 mg/kg daily) for 4 days. Mice cisplatin groups were given the same volume of vehicle (equal volume). At the end of the experience, the blood and kidneys were collected when the animals were sacrificed.

### 2.14 Biochemical analysis and histopathology

To evaluate the renal function in mice, blood samples from mice were separated by centrifugation at 1000 *g* for 15 min, and the clinical biochemistry analysis of creatinine (Crea) and urea nitrogen (BUN) in the serum was measured by commercial kits (Stanbio Laboratory).

### 2.15 ELISA analysis

We use the ELISA kits to measure the levels of tumour necrosis factor‐α (TNF‐α, EMC102a) and interleukin 6 (IL‐6, EMC004) in the serum according to the protocol of manufacturer (Neobioscience Technology Company).

### 2.16 Histological analysis

Kidney tissues were harvested after extensive perfusion with saline, fixed with 4% paraformaldehyde. Then, the paraffin‐embedded tissues were sectioned at 4 μm. After that, the sections were stained with haematoxylin and eosin (HE) and periodic Acid‐Schiff (PAS) stains before examination under a light microscope.

### 2.17 TUNEL detection

To detect the apoptosis in mice after cisplatin injection, DNA fragmentation in kidney tissues was determined by TdT‐mediated dUTP nick‐end labelling (TUNEL) assay (1,684,817; Roche Applied Science). Briefly, frozen sections were air‐dried for 30 min and incubated with 4% PFA for 20 min at room temperature; then, the sections were permeabilized with 0.1% Triton X‐100 in 0.1% sodium citrate solution and stained with the (TUNEL) assay kit for 1 h at 37°C in a dark chamber, and the nuclei were labelled by Hoechst33342 (H‐1399; Thermo Fisher Scientific). Detection of the apoptotic cells showing green fluorescence was morphologically analysed.

### 2.18 Quantitative reverse transcriptase PCR (q‐PCR)

The mRNA expression was determined by quantitative reverse transcriptase PCR using SYBR Green (1,176,102 K; Thermo Fisher Scientific) as previously described.^21^ The specific sequences of primers for different genes are described in Table S1. The relative expression of target genes was calculated according to the ΔΔCt method and normalized on ACTIN.

### 2.19 Statistical analysis

Data obtained from this study were expressed as mean ± SEM. To compare the differences between groups, we used one‐way analysis of variance (anova), followed by Tukey's test for post hoc test using GraphPad Prism software. Differences with statistical significance were set at *p* < 0.05.

## 3 RESULTS

### 3.1 MAT prevents the cisplatin‐induced cell injury

**FIGURE 1:**
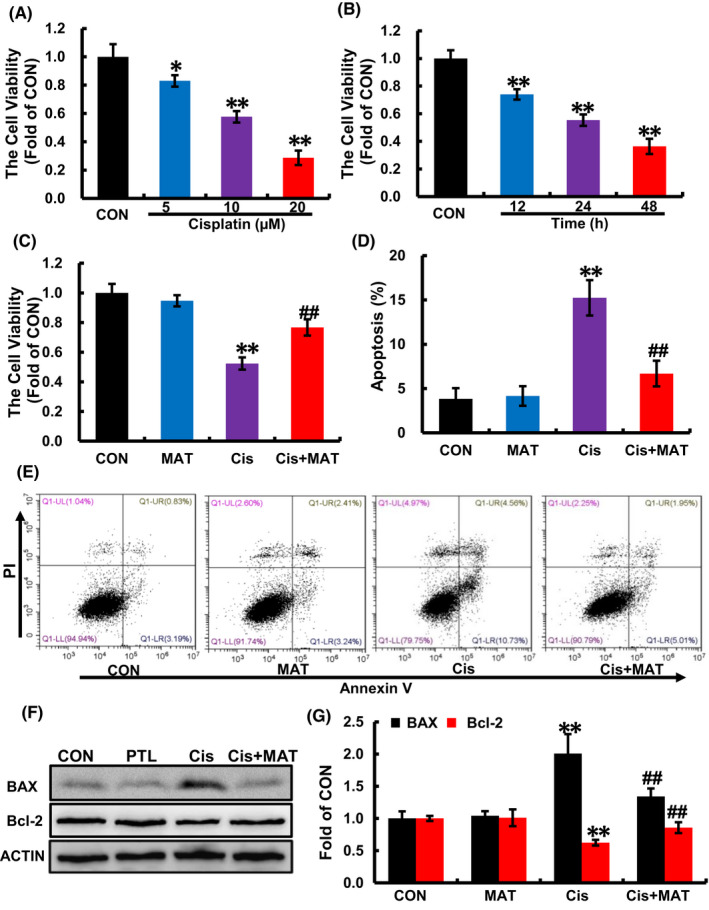
Matrine inhibits cisplatin‐induced cell injury in HK2 cells. Cells were incubated with cisplatin (Cis, 10 μM) in the present or absent of matrine (MAT, 400 μΜ). (A–C) Cell viability was measured by CCK8 assay. (D and E) For apoptosis assay, the cells were stained with Annexin V‐FITC/PI cell apoptosis detection kit and analysed by flow cytometry. (F and G) Western blotting and quantification analysis of apoptosis‐related BAX and Bcl‐2 protein levels. The data are presented as the means ± SEM (*n* = 3). Bar graphs represent means ± SEM *p < 0.05 and **p < 0.01 versus control (CON); ^**##**^ *p* < 0.01 versus cisplatin (Cis)

After stimuli of cisplatin, the cell viability was decreased in a dose‐dependent and time‐dependent manner (Figure 1A,B). To explore the effects of MAT on cisplatin‐induced cell injury, MAT (400 μΜ) was added into cisplatin‐treated HK2 cells. Compared with cisplatin group, treatment with MAT increased cell viability from 52.3% ± 0.2% to 78.6% ± 0.3% (Figure 1C). Due to the critical factor of apoptosis in cisplatin‐induced nephrotoxicity, we further determine the effect of MAT on apoptosis, and we found that the apoptosis rate was dramatically reduced in cisplatin +MAT group from 13.7% ± 0.1% to 7.2% ± 0.4% (Figure 1D,E). Moreover, MAT treatment reduced the expression of pro‐apoptotic BAX and increased the expression of anti‐apoptotic Bcl‐2 (Figure 1F,G). These dates suggested that MAT alleviated cisplatin‐induced cell injury.

### 3.2 MAT inhibits cisplatin‐induced oxidative injury and inflammation

Excessive accumulation of ROS stimulated by cisplatin plays a critical factor in cisplatin‐caused nephrotoxicity.^22^ Consistent with the observation, we found that cisplatin treatment (10 μM) for 48 h induced an 1.52 ± 0.03 fold increase in ROS level compared with that in control group; however, treatment with MAT obviously attenuated ROS content (Figure S1A,B). As a consequence of oxidative injury, we detected the endogenous antioxidants catalase (CAT) activity and the malondialdehyde (MDA) content. Treatment with MAT increased the CAT activity in comparison with the cisplatin group (Figure S1C). Moreover, after treatment with MAT, the MDA content in HK2 cells exposure with cisplatin was notably reduced (Figure S1D), indicating that MAT inhibited the oxidative injury induced by cisplatin.

Oxidative stress could cause inflammation in acute kidney injury. As the first signal, activation of nuclear transcription factor‐kappa B (NF‐κB) could drive the inflammatory response and prime the pathogenesis of kidney‐related diseases.^23^ Hence, we evaluated the effects of MAT on NF‐κB activation via immunofluorescence. As expected, cisplatin stimuli induced nuclear translocation of NF‐κB and phosphorylation of NF‐κB, suggesting its activation (Figure S2A,B). However, incubation with MAT blocked the activation of NF‐κB (Figure S2A,B). As a consequence, the elevated mRNA levels of the pro‐inflammatory cytokines, including IL‐1β, IL‐6 and TNF‐α induced by cisplatin, as determined by RT‐PCR, were reduced after MAT treatment (Figure S2C–E). Overall, these findings suggested that MAT treatment inhibited cisplatin‐induced inflammation via inactivation of NF‐κB pathway.

### 3.3 MAT reverses cisplatin‐induced loss of mitochondrial function in HK2 cells

**FIGURE 2:**
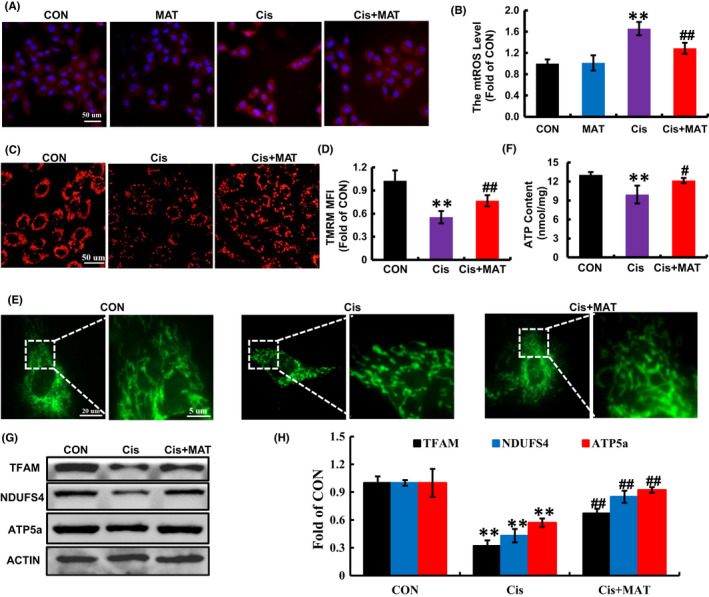
Matrine treatment improves mitochondrial function in cisplatin‐treated HK2 cells. (A and B) Mitochondrial ROS (mtROS) was determined by MitoSOX Red. (C and D) The mitochondrial membrane potential (*Δψm*) was detected by TMRM staining. (E) Representative immunofluorescence of mitochondrial morphology in HK2 cells, loaded with Mito‐tracker‐green (100 nM). (F) The ATP content in cells was detected by commercial ATP bioluminescence assay kit. (G and H) Western blotting and quantification analysis of mitochondria biogenesis‐related proteins (TFAM) and mitochondrial electron transport chain complex related proteins (NDUFS4 and ATP5a). Data represent 3 independent experiments, and are presented as the means ± SEM. **p < 0.01 versus CON group; ^**#**^ *p* < 0.05 and ^**##**^ *p* < 0.01 versus Cis group

As previously reported, besides the ATP supply via oxidative phosphorylation (OXPHOS), ROS, as a by‐production, are mainly generated in mitochondria.^24^ In our study, cisplatin increased excessive accumulation of mtROS (Figure 2A,B) and mitochondrial depolarization (Figure 2C,D). Conversely, MAT treatment significantly reversed all of these changes (Figure 2A–D). Moreover, Mito‐Tracker Green staining showed that MAT treatment could suppress the mitochondrial fragmentation compared with discontinuous and fragmented mitochondria in cisplatin‐stimulated HK2 cells (Figure 2E). As a consequence of improved mitochondria, the content of ATP in cisplatin‐induced HK2 cells was elevated from 9.94 ± 1.42 nmol/mg protein to 12.14 ± 0.41 nmol/mg protein (Figure 2F). Furthermore, MAT increased the protein expression of TFAM, NDUFS4 and ATP5a, which were associated with mitochondrial biogenesis and mitochondrial electron transport chain complex (Figure 2G,H). Taken together, the observations indicated that MAT restored mitochondrial function in cisplatin‐induced HK2 cells.

### 3.4 The protective role of MAT in cisplatin‐induced mitochondrial dysfunction is relied on SIRT3/OPA1

**FIGURE 3:**
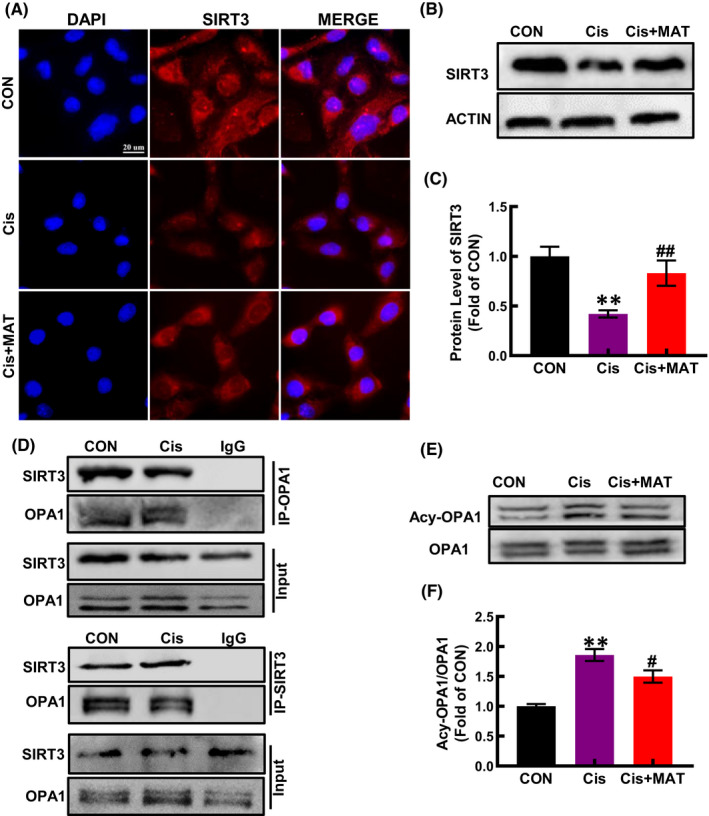
Matrine increases SIRT3 expression and its deacetylation activity. (A) Representative immunofluorescence of SIRT3 in HK2 cells with anti‐SIRT3 (red) and DAPI (blue). (B and C) The expression of SIRT3 was determined by Western blotting. (D) Cell lysates were immunoprecipitated (IP) with an anti‐SIRT3 or an anti‐OPA1 antibody and then immunoblotted (IB) with OPA1 and SIRT3 antibodies. Anti‐IgG antibody as a negative control. (E and F) Acetylation of OPA1 in HK2 cells with MAT treatment. The data are presented as the means ± SEM (*n* = 3). **p < 0.01 compared with CON group; ^**#**^ *p* < 0.05 and ^**##**^ *p* < 0.01 compared with Cis group

Since SIRT3 is a critical regulator of mitochondrial integrity in AKI,^25^ we detected the expression of SIRT3 in HK2 cells and found that the level of SIRT3 was reduced under cisplatin stimuli, while increased after MAT treatment (Figure 3A–C). SIRT3, a member of NAD+ − dependent deacetylases, deacetylates many mitochondrial proteins. OPA1 is the master regulator of mitochondrial fusion, and its activity is negatively associated with the acetylation level. Interestingly, we found that SIRT3 could coimmunoprecipitate with OPA1 (Figure 3D). Besides, along with the increased SIRT3 expression, the acetylation of OPA1 induced by cisplatin was suppressed (Figure 3E,F). In addition to the OPA1, MFN1 is responsible for mitochondrial outer membrane fusion,^26^ we found that cisplatin stimuli decreased the expression of MFN1 in HK2 cells, while no significant change was observed after MAT treatment (Figure S3A). As we all know, the regulation of mitochondrial dynamics is a complicated process involving various dynamin‐related GTPases, which maintain a balance between mitochondrial fission and fusion. Mitochondria fission is catalysed by DRP1, which is recruited by binding to its receptor to form a functional complex anchored to the outer membrane of mitochondria (MOM), and the process was rely on phosphorylation of DRP1 at serine 656 residue.^26^ Here, the phosphorylation of DRP1 was induced by cisplatin, while MAT treatment has no effect on that (Figure S3B), suggesting that MAT‐mediated inhibition of mitochondrial fragmentation was independent on mitochondrial fission. Thus, we conclude that MAT coffered improvement of mitochondrial function may be due to activation of the SIRT3/OPA1 pathway.

**FIGURE 4:**
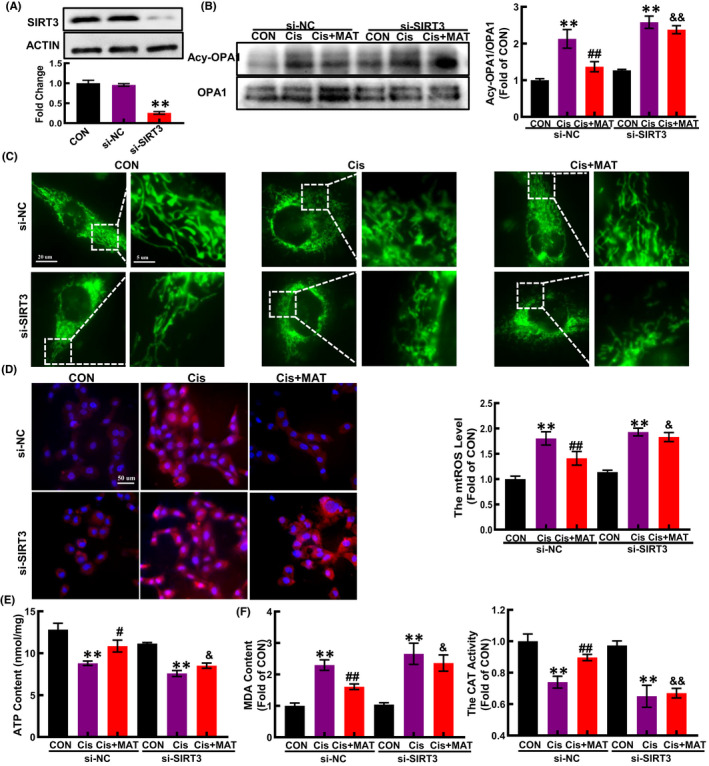
Silencing of SIRT3 partially abolishes the protective effects of MAT on mitochondrial dysfunction and oxidative injury induced by cisplatin in HK2 cells. Cells were transfected with control siRNA (si‐NC) or SIRT3 siRNA (si‐SIRT3) for 6 h and treated with cisplatin in the presence or absence of MAT for 48 h. (A) The expression of SIRT3 was determined by Western blotting. (B) Acetylation of OPA1 in HK2 cells. (C) Representative immunofluorescence of mitochondrial morphology in HK2 cells. The mtROS level (D) and the ATP content (E) in HK2 cells. (F) The MDA content and CAT activity in cells were measured by chemical kit. The data are presented as the means ± SEM (*n* = 3). **p < 0.01 compared with CON group; ^**#**^ *p* < 0.05 and ^**##**^ *p* < 0.01 compared with Cis group; ^&^ *p* < 0.05 and ^&&^ *p* < 0.01 compared with Cis + MAT group

**FIGURE 5:**
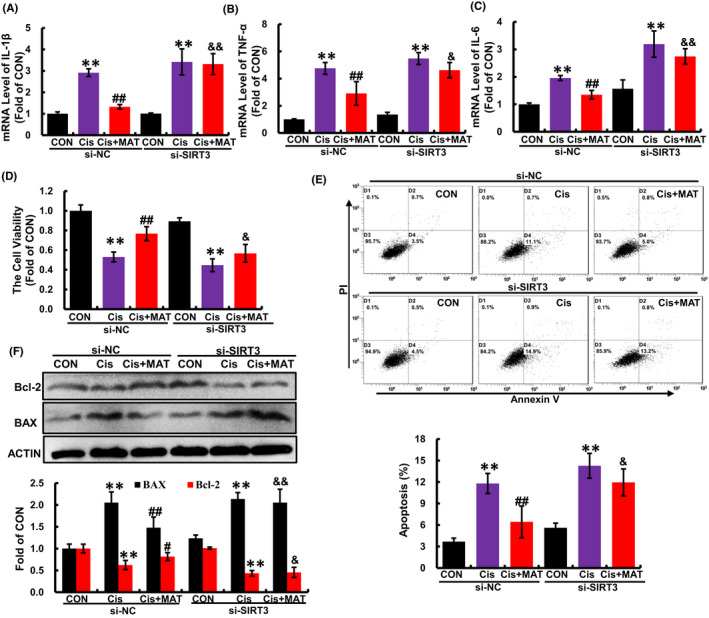
Matrine‐alleviated inflammation and cell injury in cisplatin‐treated HK2 cells was dependent on SIRT3. (A–C) The mRNA levels of IL‐1β, TNF‐α and IL‐6 in SIRT3‐knockdown HK2 cells. The cell viability (D) and apoptosis (E) were determined. (F) The expression of BAX and Bcl‐2 was assessed by Western blotting. The data are presented as the means ± SEM (*n* = 3). **p < 0.01 compared with CON group; ^**##**^ *p* < 0.01 compared with Cis group; ^&^ *p* < 0.05 and ^&&^ *p* < 0.01 compared with Cis + MAT group

**FIGURE 6:**
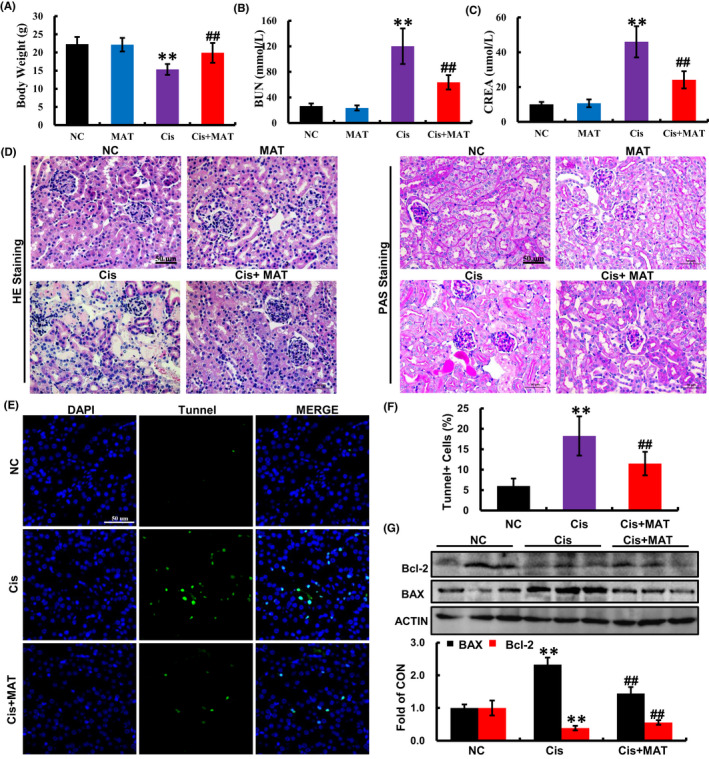
Matrine treatment alleviates nephrotoxicity and apoptosis induced by cisplatin in mice. (A) The body weight, (B) the BUN level and (C) the serum CREA at 4 days after cisplatin injection. (D) Morphological changes of kidney tissues from different group were measured by haematoxylin and eosin and PAS staining. Arrows indicate acute tubular necrosis; pound signs indicate vacuolization. (E and F) The apoptosis in kidney tissues from mice was measured by TdT‐mediated dUTP nick‐end labelling (TUNEL) assay, and the TUNEL‐positive apoptotic cells were quantified by Image‐J software. (G) Western blotting and quantification analysis of apoptosis‐related BAX and Bcl‐2 protein levels. All data are quantified from 6 mice/group and shown as the means ± SEM. **p < 0.01 versus normal control (NC); ^**##**^ *p* < 0.01 versus Cis

To further verify whether the alleviation of mitochondrial dysfunction by MAT was dependent on SIRT3, HK2 cells were transfected with si‐SIRT3. As shown in Figure 6, after knockdown of SIRT3 (Figure 4A), the acetylation of OPA1 was substantially increased compared with MAT group (Figure 4B). In addition, MAT‐mediated inhibition of mitochondrial fragmentation and mtROS excessive accumulation were significantly blocked (Figure 4C,D). Moreover, the ATP content in MAT‐treated cells was decreased when the SIRT3 was knockdown (Figure 4E). Due to the counteraction of improved mitochondrial function, the MDA content in SIRT3‐knockdown HK2 cells was elevated compared with MAT‐treated cells, while the CAT activity was reduced (Figure 4F). Additionally, the mRNA levels of pro‐inflammatory factors (IL‐1β, IL‐6 and TNF‐α) were increased compared with the si‐NC cells (Figure 5A–C). Consequently, MAT failed to rescue the cell injury induced by cisplatin when SIRT3 was knockdown, characterized as the reduced cell viability, increased cell apoptosis rate (from 5.8% ± 0.2% to 14.5% ± 0.6%) and elevated expression of BAX (Figure 5D–F). These findings indicated that the protective effects of MAT in cisplatin‐induced HK2 cells were relied on the activation of SIRT3.

### 3.5 MAT attenuates renal injury and apoptosis in cisplatin‐induced AKI mice

When evaluating the efficacy of the MAT, toxicity and safety should be initially taken into account. Based on the previous study, MAT (>10 mg/kg/day) existed hepatotoxicity and neurotoxicity in mice.^27^ For the safety of MAT treatment, we choose the concentration (<10 mg/kg/day) in our study. As shown in Figure S4A,B, we found that MAT treatment at doses of 2.5, 5 and 10 mg/kg/day have no effects on liver function parameters (ALT and AST) and kidney function parameters (BUN and CREA). Moreover, there was no significant difference in pathological changes of liver and kidney between MAT and control group (Figure S4 C and D), suggesting that MAT did not induce hepatotoxicity and nephrotoxicity in mice. Four days after cisplatin administration, the mice developed severe renal dysfunction as indicated by the reduction in body weight and increase in BUN and CREA compared with the normal mice (Figure 6A–C). To evaluate the effect of MAT on renal injury caused by cisplatin, MAT was administrated daily for four continuous days. Here, in comparison with AKI mice, the serum levels of BUN and CREA were decreased to 63.67 ± 11.25 mM and 24.17 ± 4.91 μM, respectively, after 5 mg/kg MAT treatment (Figure 6A–C). In addition, 10 mg/kg MAT did not further potentiate the protective effect on kidney compared with the 5 mg/kg/day group, indicating that 5 mg/kg MAT in mice already reached its maximum benefit in this disease model (Figure S5). Therefore, 5 mg/kg/day of MAT was used in the following experiments. Consistently, histological analysis revealed that extensive tubular injury observed in AKI mice, including renal tubular necrosis, formation of tubular casts, cytoplasmic vacuoles and renal infiltration of inflammatory cells were markedly attenuated (Figure 6D). These findings demonstrated that MAT attenuated cisplatin‐induced AKI in mice.

The apoptosis in the kidneys of AKI mice was measured by TUNEL staining. Compared with the normal mice, the number of TUNEL‐positive cells was significantly increased in the kidneys of cisplatin‐treated mice, which was strikingly reduced by MAT administration (Figure 6E,F). Consistent with the observation, the increased pro‐apoptotic BAX and decreased anti‐apoptotic Bcl‐2 were reversed by MAT administration compared with the cisplatin group (Figure 6G). These findings indicated that MAT ameliorated cisplatin‐induced apoptosis in vivo.

### 3.6 MAT reduces inflammatory responses in AKI mice

As we all know, pro‐inflammatory cytokines and mediators were associated with renal injury in cisplatin‐caused AKI mice.^28^ Therefore, we explored the role of MAT on inflammation, and we found that compared with the AKI mice, the increased serum levels of TNF‐a and IL‐6 were remarkably decreased after MAT treatment (Figure S6A,B). Meanwhile, by RT‐PCR, we confirmed that the mRNA levels of TNF‐a, IL‐1β and IL‐6 in kidney from AKI were strikingly increased, while reduced in Cisp+MAT mice (Figure S6C–E). Consistent with the observation in vitro, MAT administration also inhibited the phosphorylation of NF‐κB (Figure S6F). Our data indicated that MAT administration could suppress NF‐κB‐mediated systemic inflammation in cisplatin‐induced AKI mice.

### 3.7 MAT activates SIRT3/OPA1 axis and rescues renal mitochondrial dysfunction in AKI mice

**FIGURE 7:**
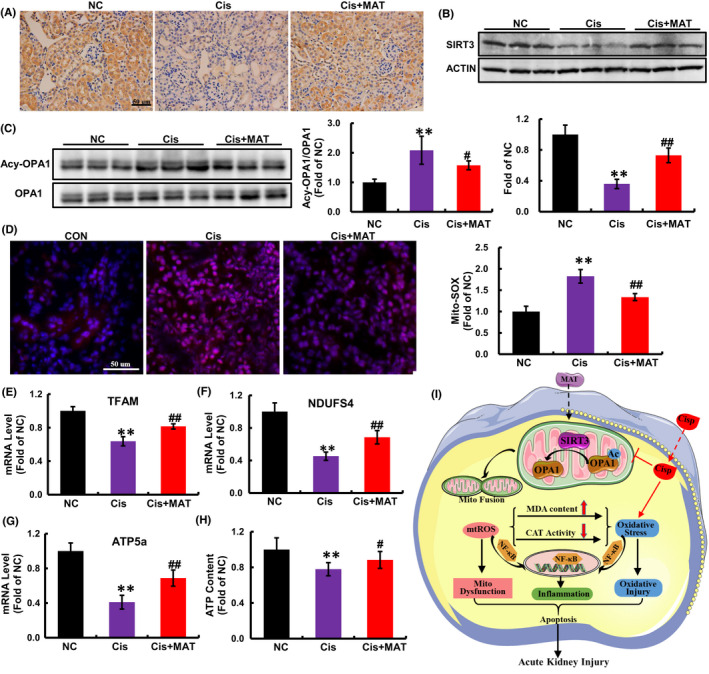
Matrine treatment activates SIRT3/OPA1 axis and rescues kidney mitochondrial function in cisplatin‐induced AKI mice. (A) Representative immunohistochemical staining of SIRT3 in mouse kidney sections among different groups. (B and C) SIRT3 expression and acetylation of OPA1 in mouse kidney sections were detected by Western blotting (*n* = 3). (D) mtROS was determined by MitoSOX Red staining in mouse kidney sections. (E–G) The mRNA levels of TFAM, NDUFS4 and ATP5a. (H) The ATP level in kidney tissues from mice. (I) Schematic diagram of mechanism that MAT prevented cisplatin‐induced cell injury and renal failure. The data are presented as the means ± SEM. All data are quantified from 4 mice/group and shown as the means ± SEM. **p < 0.01 versus the NC; ^**#**^ *p* < 0.05 and ^**##**^ *p* < 0.01 versus Cis

Consistent with in vitro findings, we found that the reduced expression of SIRT3 induced by cisplatin injection was elevated after MAT treatment (Figure 7A,B). Deacetylase activity is a major function of SIRT3. As a substrate of SIRT3, the acetylated OPA1 in AKI mice was dramatically decreased after MAT administration (Figure 7C), indicating that MAT activated SIRT3/OPA1 axis in AKI mice. Moreover, the mtROS levels in the kidney tissues were dramatically decreased in MAT‐treated mice (Figure 7D). Moreover, compared with the AKI mice, the mRNA levels of TFAM, NDUFS4 and ATP5a were increased (Figure 7E–G). As a result of improved mitochondria, the ATP content in AKI mice was increased after MAT treatment (Figure 7H). Collectively, the data confirmed that MAT treatment could activate SIRT3/OPA1 axis and improve mitochondrial function in cisplatin‐induced AKI mice.

## 4 DISCUSSION

Matrine has been demonstrated to inhibit oxidative injury and inflammatory response in CCl4 model of liver injury.^15^ Moreover, one research has demonstrated that the MAT alleviated renal damage in adriamycin‐induced nephropathy rat model.^29^ However, whether MAT could inhibit renal injury and the underlying mechanisms in cisplatin‐caused AKI mice was unclear. In current study, we demonstrated that MAT alleviated renal injury and apoptosis in AKI mice induced by cisplatin. Moreover, the renal protection of MAT was associated with the improvement of mitochondrial function via the SIRT3/OPA1 pathway.

Once cisplatin injection in vivo, it was primarily detained in the renal tubules, which drove the oxidative injury, mitochondria dysfunction, renal infiltration of inflammatory cells and eventually led to cell death in AKI mice.^30^ As previous studies proved, ROS‐mediated oxidative stress, including elevated MDA content and decreased activity of endogenous antioxidant enzymes (GSH‐Px and catalase superoxide dismutase), could induce inflammation by activating toll‐like receptors (TLRs) and the NLRP3 inflammasome pathway, which contributes to the development of renal damage induced by cisplatin.^28^, ^31^, ^32^ Moreover, in addition to the inflammation, Haohao Shi et al.^33^ have demonstrated that cisplatin treatment activated caspase mitochondrial cascade apoptosis in the kidney, while suppressing the imbalance of oxidative stress via activation of SIRT1/PGC‐1α and inhibition of MAPK pathway could reverse mitochondrial cascade apoptosis and alleviated kidney injury in AKI. Similar with the previous studies, MAT administration inhibited the oxidative injury and apoptosis comparing with the AKI mice in our study.

Mitochondria plays essential roles in regulation of energy metabolism, oxidative stress response to stress stimuli.^34^, ^35^ During kidney damage, ROS are mainly generated from the electron transport chain in the mitochondria.^36^ In recent studies, emerging evidence showed that cisplatin injection induces excessive production of mtROS by impairing mitochondrial electron transport chain components, suggesting that targeting mitochondrial preservation may be a promising strategy for AKI.^37^, ^38^, ^39^ Consistent with previous studied, we found that the mitochondrial ROS in kidney form AKI mice was dramatically increased. In addition, a recent research has found that MAT treatment could inhibit hepatic inflammation and lipid peroxidation, which owe to the restoration of mitochondrial dysfunction in non‐alcoholic fatty liver mice model, suggesting that MAT inhibits mitochondrial function and subsequent inflammation.^40^ Similar with the results, along with the reduction in mtROS levels, the mRNA levels of mitochondrial electron transport chain components and ATP content in kidney from AKI mice were increased after MAT administration, indicating that MAT alleviated impaired mitochondria in AKI mice.

Mitochondrial dysfunction and subsequent release of the mtDNA is a critical regulator of systemic inflammation in AKI models.^41^ Consistent with the observation, our study found that cisplatin injection induced systemic inflammation in mice, as characterized by the elevated TNF‐α and IL‐6, whereas suppressed by MAT in vivo. Increasing evidence suggested that during AKI process, activation of NF‐κB pathway prime assembly of inflammasome, which subsequently lead to the release of mature IL‐1β.^42^, ^43^ Here, our study showed that MAT treatment could inhibit the nuclear translocation of NF‐κB, indicating that MAT conferred inhibition of inflammation may be rely on the suppression of NF‐κB/NLRP3 pathway. As noted, we speculate that the renal‐protective role of MAT in AKI mice induced by cisplatin may be dependent on the inhibition of mitochondrial dysfunction and subsequent systemic inflammation.

SIRT3, mainly located in mitochondria, exhibits global mitochondrial lysine deacetylase activity, and increasing evidence verified the key role of SIRT3 in mitochondrial energy metabolism. Previous studies have demonstrated that the expression of SIRT3 in kidney tissue was significantly decreased in cisplatin‐induced AKI mice, while therapeutic strategies to restore SIRT3 expression could prevent tubular injury and improve renal function.^25^, ^44^ Keep in line with the observation, the reduced expression of SIRT3 was also confirmed in our study. Moreover, not only the expression, but also deacetylase activity of SIRT3 were significantly increased after MAT treatment.

OPA1, the GTPase anchored to the inner membrane (IM) of mitochondria, exists in multiple long and short isoforms and performs multiple functions. Besides being an obligatory protein for the IM fusion, OPA1 is also involved in maintaining crista structure and protecting cells from death stimuli.^45^ In previous study, researchers have confirmed that pathological stress could induce acetylation of OPA1, which result in the reduction in its GTPase activity. In addition to matrix, SIRT3 is also reported to be presented at mitochondrial IM and crista structures. In addition, in a further research, the authors confirmed that SIRT3 bound directly to OPA1 and activates it by deacetylation in vitiligo melanocytes under oxidative stress,^46^ suggesting that the OPA1 is a direct target of SIRT3. Keep in line with the observation, we found that due to elevated activity of SIRT3, MAT treatment protected against cisplatin‐induced mitochondrial fragmentation via deacetylation of OPA1. In addition, Wang et al. have demonstrated that MAT could effectively alleviate EAE, a certain extent, by protecting stressed oligodendrocytes (OLGs) through enhancing mitophagy.^12^ Mitophagy, a specific autophagic pathway, could maintain the mitochondria homeostasis via selective scavenging of impaired mitochondria entrapped in autophagosomes.^4^ Moreover, several studies have demonstrated that SIRT3 could activate mitophagy and subsequently alleviate mitochondrial dysfunction in senile osteoporosis, diabetic cardiomyopathy and hepatic ischemia–reperfusion.^47^, ^48^, ^49^ Based on the improvement of mitochondrial function of MAT in our study, we speculate that besides to deacetylation of OPA1, SIRT3‐activated mitophagy may play a particular role in renal‐protective effect of MAT in AKI mice and also need further exploration.

In conclusion, our study demonstrated the MAT treatment could inhibit renal injury in AKI mice and cytotoxicity in HK2 cells induced by cisplatin. Furthermore, we also found that after MAT administration, the cisplatin‐induced oxidative injury and systemic inflammation were significantly suppressed, which was associated with SIRT3/OPA1 axis mediated improvement of mitochondrial dysfunction (Figure 7I). However, as an adjuvant therapy, the clinical use of MAT to attenuate cisplatin‐caused nephrotoxicity needs further studies.

## AUTHOR CONTRIBUTIONS

**Lu Yuan:** Data curation (equal); investigation (equal); methodology (equal); validation (equal); writing – original draft (equal); writing – review and editing (equal). **Jingchao Yang:** Methodology (equal); validation (equal). **Ying Li:** Data curation (equal); supervision (equal). **Longhui Yuan:** Data curation (equal); validation (supporting). **Fei Liu:** Writing – review and editing (supporting). **Yujia Yuan:** Project administration (equal); supervision (equal). **Xiaochi Tang:** Project administration (lead); supervision (lead); writing – review and editing (lead).

## CONFLICT OF INTEREST

No competing financial interests exist.

## Supporting information


Figure S1

Figure S2

Figure S3

Figure S4

Figure S5

Figure S6

Table S1

## Data Availability

The data that support the findings of this study are available from the corresponding author upon reasonable request.
