# Supplementary figures and images for: Matrine alleviates cisplatin‐induced acute kidney injury by inhibiting mitochondrial dysfunction and inflammation via SIRT3/OPA1 pathway
Source: J Cell Mol Med. 2022 Jun 1;26(13):3702–15. doi: 10.1111/jcmm.17398 (PMC9258713; doi:10.1111/jcmm.17398)

Fig. S1

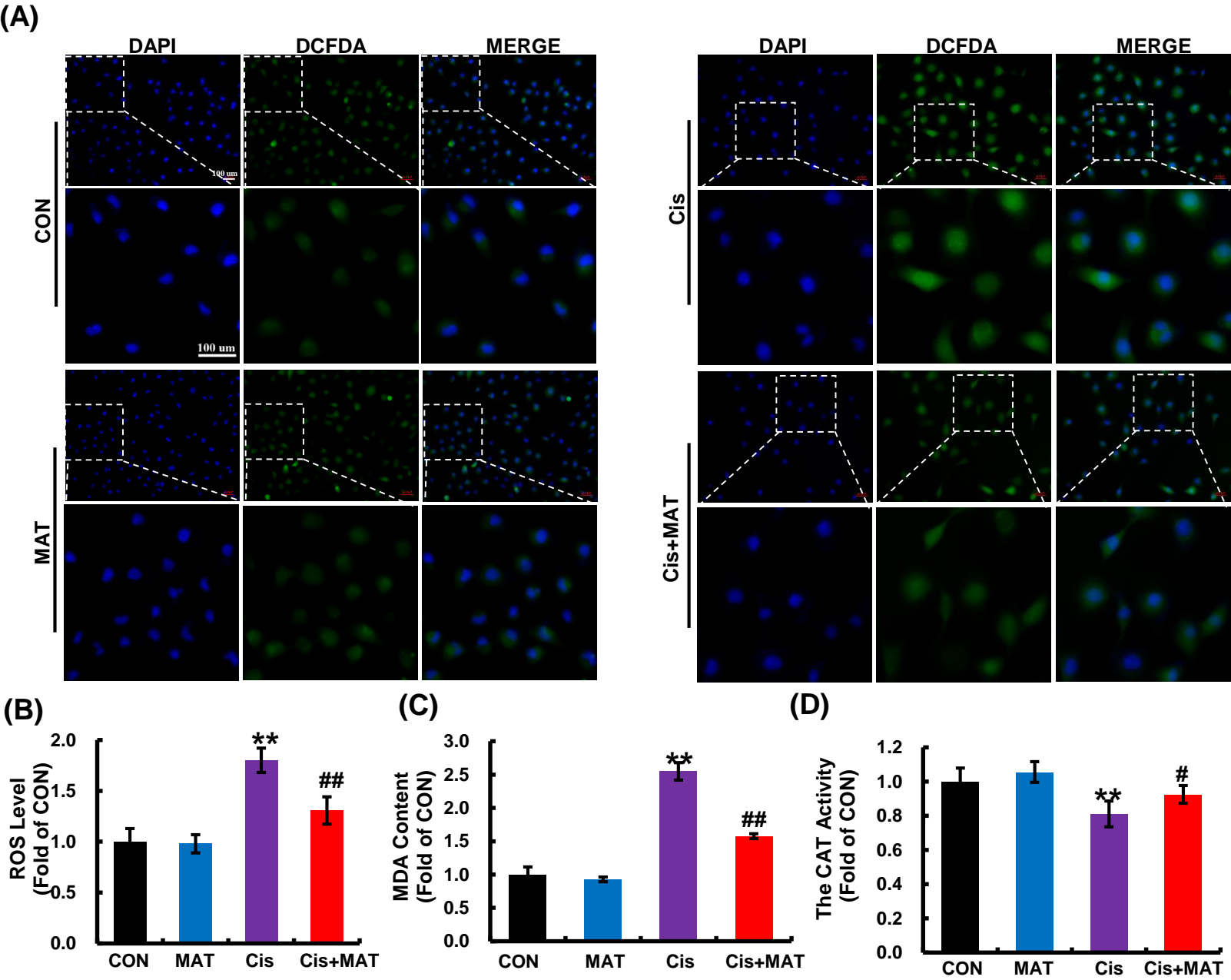

Supplement: Supplementary file 1 — Figure S1 [file JCMM-26-3702-s006.pdf]

Fig. S2

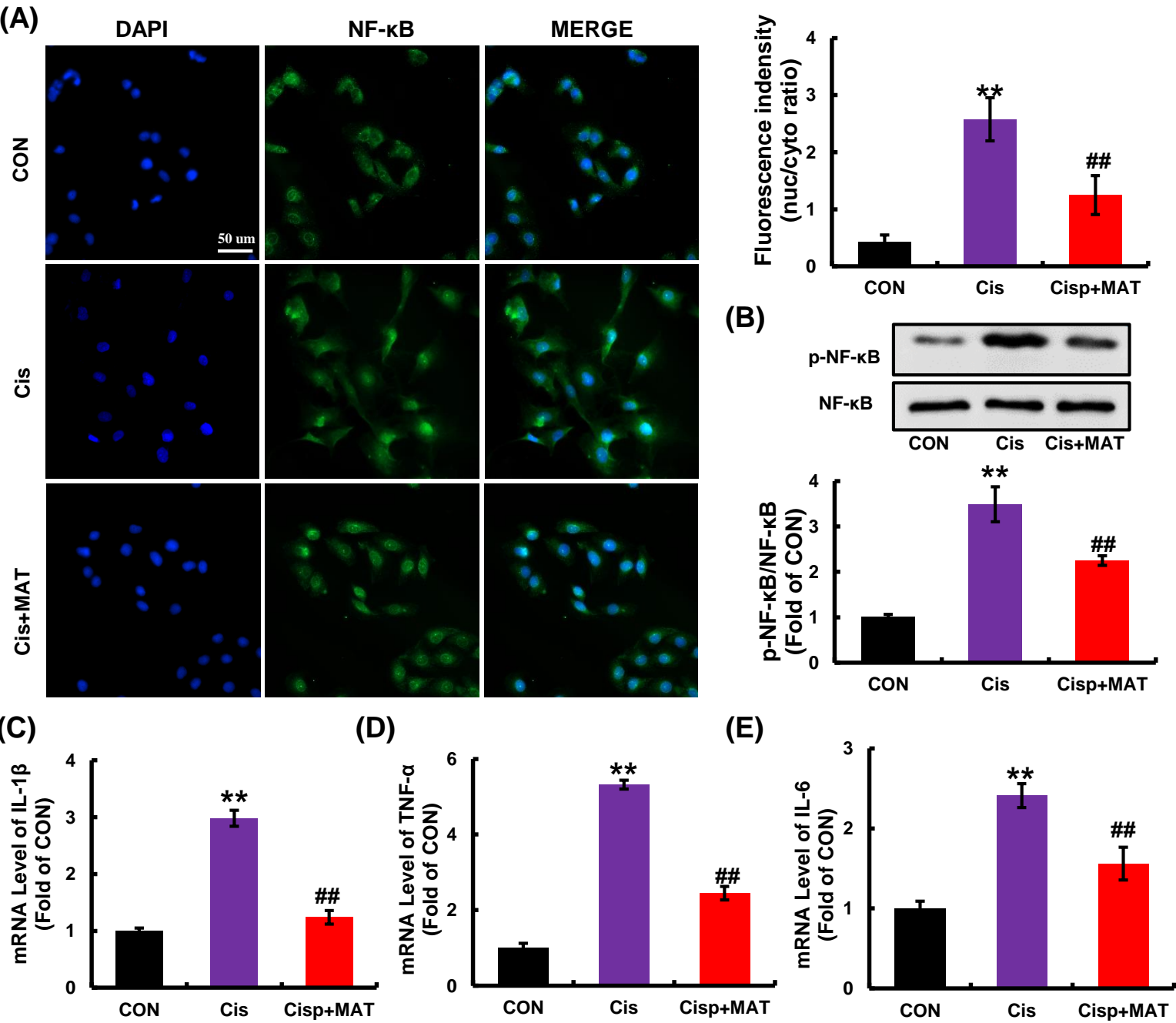

Supplement: Supplementary file 2 — Figure S2 [file JCMM-26-3702-s001.pdf]

Fig. S3

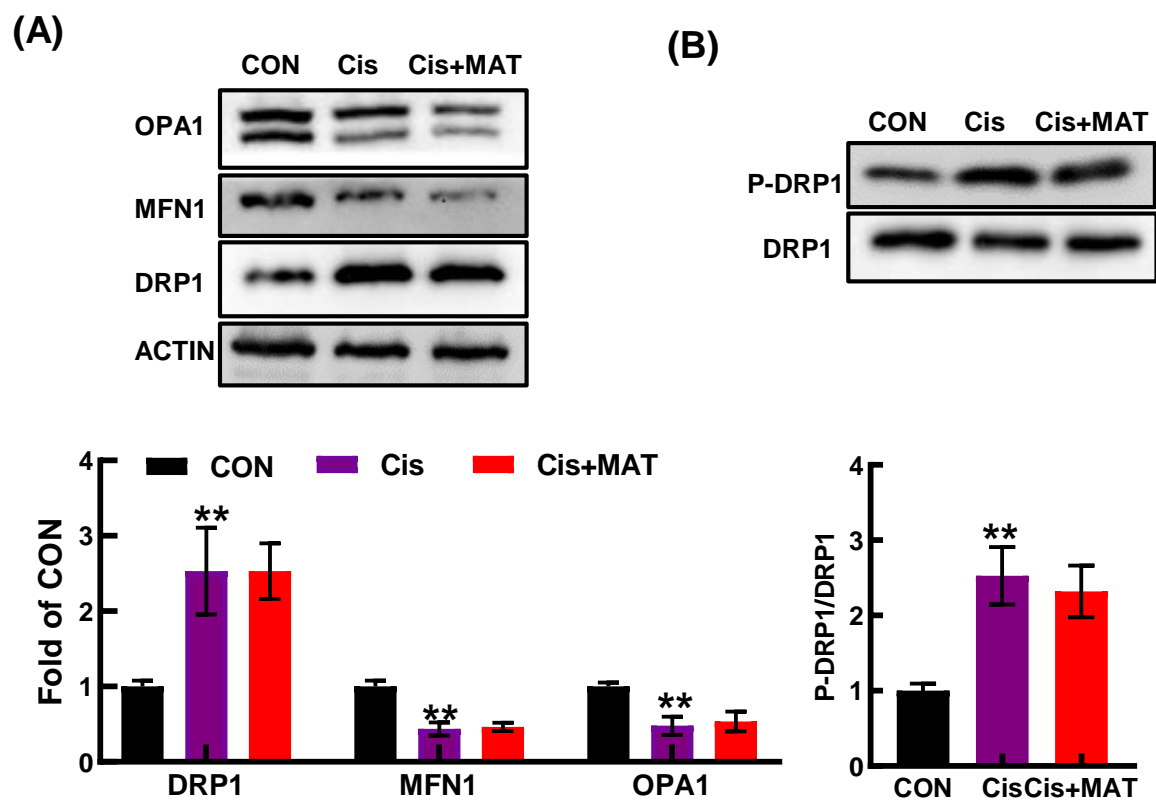

Supplement: Supplementary file 3 — Figure S3 [file JCMM-26-3702-s003.pdf]

Fig. S4

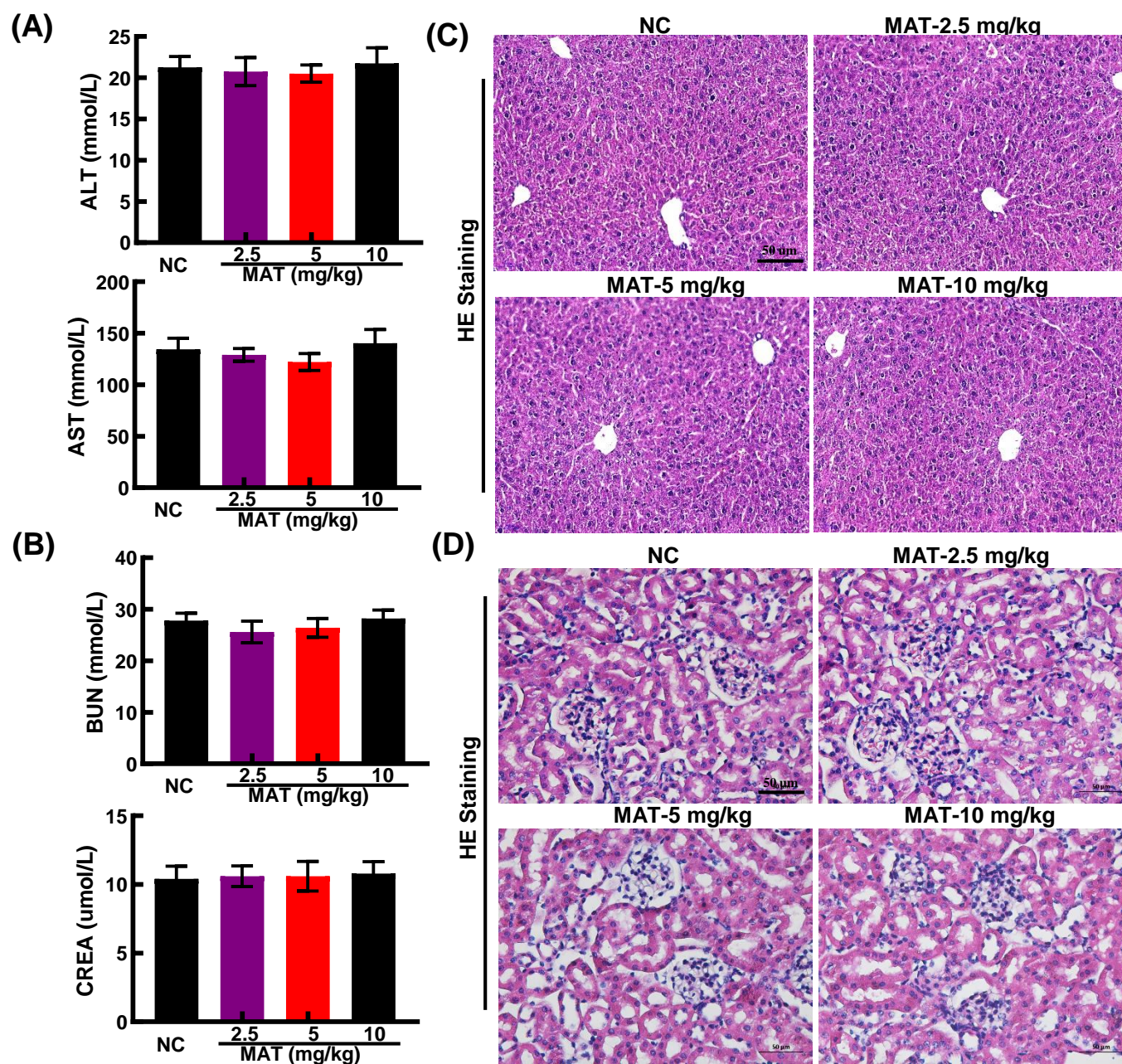

Supplement: Supplementary file 4 — Figure S4 [file JCMM-26-3702-s002.pdf]

**Fig. S5**

**(A)**

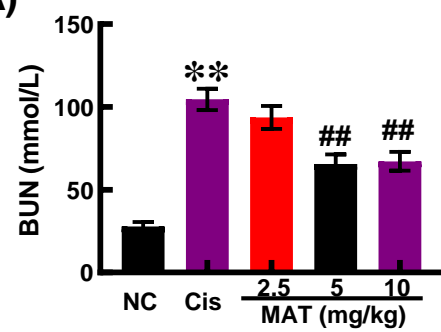

**(B)**

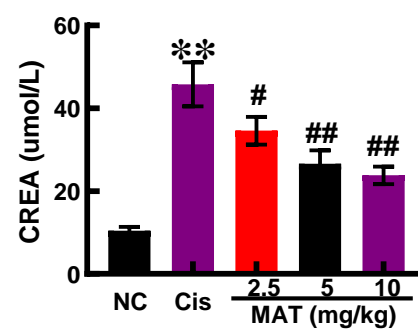

Supplement: Supplementary file 5 — Figure S5 [file JCMM-26-3702-s007.pdf]

Fig. S6

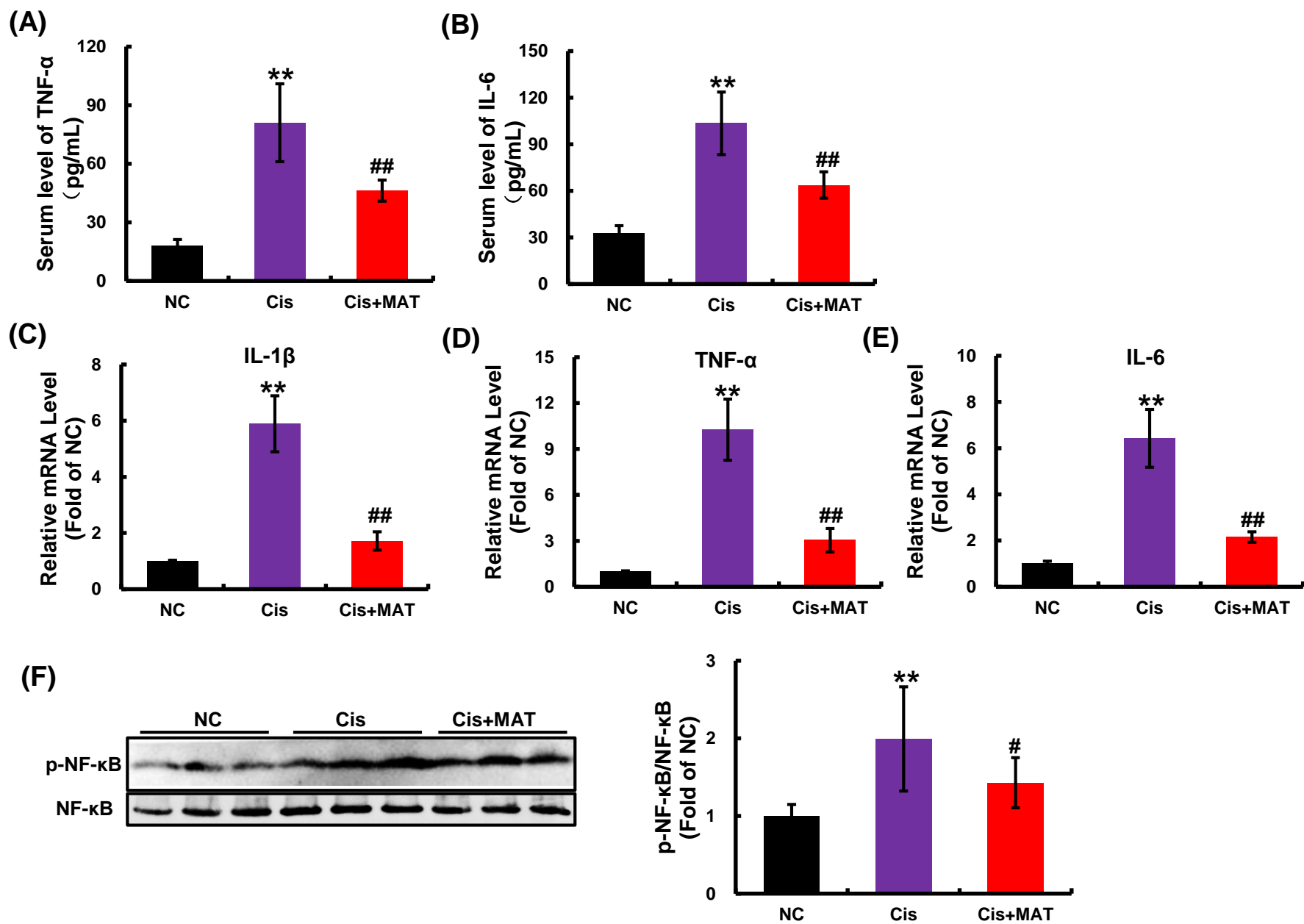

Supplement: Supplementary file 6 — Figure S6 [file JCMM-26-3702-s004.pdf]
