# Supplementary material for: Matrine alleviates cisplatin‐induced acute kidney injury by inhibiting mitochondrial dysfunction and inflammation via SIRT3/OPA1 pathway
Source: J Cell Mol Med. 2022 Jun 1;26(13):3702–15. doi: 10.1111/jcmm.17398 (PMC9258713; doi:10.1111/jcmm.17398)
Supplement: Supplementary file 7 — Table S1 [file JCMM-26-3702-s005.docx]

| TFAM-F | GGAGGCAAAGGATGATTCGG |
| --- | --- |
| TFAM-R | TCGTCCAACTTCAGCCATCT |
| NDUFS4-F | CAAACCTCCTATCAGCCATCC |
| NDUFS4-R | AGCGAAGAATCGGGTCAAG |
| ATP5a-F | TTTGCTGGTGTTGGTGAGAG |
| ATP5a-R | GGTGGTTCGTTCATCTGTCC |
| IL-1β-F | CACCTCTCAAGCAGAGCACAG |
| IL-1β-R | GGGTTCCATGGTGAAGTCAAC |
| TNFα-F | ACGGCATGGATCTCAAAGAC |
| TNFα-R | AGATAGCAAATCGGCTGACG |
| IL-6-F | CTGCAAGAGACTTCCATCCAG |
| IL-6-R | AGTGGTATAGACAGGTCTGTTGG |
| ACTIN-F | AAGGCCAACCGTGAAAAGAT |
| ACTIN-R | GTGGTACGACCAGAGGCATAC |

**Table S1.** **The specific sequences of primers for different genes**
